# Melatonin Alleviates Acute Kidney Injury by Inhibiting NRF2/Slc7a11 Axis-Mediated Ferroptosis

**DOI:** 10.1155/2022/4776243

**Published:** 2022-08-08

**Authors:** Yue-bo Huang, Ling Jiang, Xue-qi Liu, Xian Wang, Li Gao, Han-xu Zeng, Wei Zhu, Xue-ru Hu, Yong-gui Wu

**Affiliations:** ^1^Department of Nephropathy, The First Affiliated Hospital of Anhui Medical University, Hefei, Anhui 230022, China; ^2^The Center for Scientific Research of Anhui Medical University, Hefei, Anhui 230022, China

## Abstract

Acute kidney injury (AKI) is still a puzzling clinical problem; its pathophysiology is not completely understood. Up to now, an effective treatment for AKI is lacking. Ferroptosis is a novel form of regulated cell death characterized by the lethal accumulation of lipid hydroperoxides that are dependent on iron and reactive oxygen species and mitochondrial dysfunction. Recently, ferroptosis was shown to play a vital role in AKI such as ischemia-reperfusion kidney injury and folic acid-induced AKI. Melatonin (MT) is an antioxidant that regulates the sleep-wake cycle. While the therapeutic effect of melatonin on AKI has been reported, its mechanism for the treatment of renal ferroptosis remains unclear. We found that melatonin treatment significantly alleviated the serum biochemistry index and histopathological alterations *in vivo* AKI models induced by bilateral renal artery ischemia reperfusion and folic acid in mice. Ferroptosis induced by hypoxia and reoxygenation or erastin (Era) in mouse tubular epithelial cells (MTEC) was also rescued by melatonin treatment. RNA sequence analysis of ferroptosis-related genes showed that melatonin affects oxidative stress responses by inhibiting hypoxia and reoxygenation- (HR-) mediated downregulation of *NRF2* and upregulation of *Slc7a11* in MTEC. Specific knockdown of NRF2 increased the sensitivity of cells to ferroptosis, and melatonin failed to protect against ferroptosis in the HR condition. Together, our data indicate that melatonin prevents ferroptosis in AKI by acting on the NRF2/Slc7a11 axis.

## 1. Introduction

Acute kidney injury (AKI) is a common critical illness characterized by a rapid increase in serum creatinine and the rapid decrease in glomerular filtration rate and a decrease in urine output and anuria [1]. Although the pathophysiology of AKI is not fully understood, AKI is mainly caused by multiple factors, including ischemia-reperfusion injury; nephrotoxic drugs such as methotrexate, cisplatin, and proton pump inhibitors; sepsis; and urinary tract obstruction [2]. Previous studies on AKI have mainly focused on necroptosis [3], autophagy [4], or apoptosis [3]; however, increasing evidence [5–10] suggests that ferroptosis plays a prominent role in AKI. Ferroptosis is a novel form of regulated cell death first proposed in 2012 and is characterized by the iron- and reactive oxygen species- (ROS-) dependent lethal accumulation of lipid hydroperoxides [11]. Several studies have reported that ferroptosis occurs in cancer, brain, heart, and kidney diseases [12]. Some studies have also shown that ferroptosis is a promising therapeutic target, especially in diseases characterized by kidney tubular necrosis. The inactivation of GPX4, the key ferroptosis regulator, can trigger ferroptosis in kidney tubular cells and induce acute renal failure, while the ferroptosis inhibitor, ferrostatin-1 (Fer-1), can prevent morphological changes in synchronized tubular cell death [13]. It has also been reported that ferroptosis plays an important role in nephrotoxic folic acid-induced AKI, further suggesting that ferroptosis is a potential therapeutic target for AKI [10].

The small molecules, Fer-1 and liproxstatin (Lip-1), are specific ferroptosis inhibitors that are widely used *in vitro* and *in vivo*. However, despite overwhelming evidence of their therapeutic potential, the application of Fer-1 or Lip-1 in clinical transformation is limited due to their instability *in vivo*. Thus, more economical and effective drugs need to be developed for treating ferroptosis in a clinical setting. Melatonin (MT), a pineal hormone, is an effective antioxidant that has beneficial effects on many pathological conditions, including cognition, infection, diabetic nephropathy, and AKI [14, 15]. Melatonin was reported to attenuate cisplatin-induced AKI through the dual inhibition of apoptosis and necroptosis [3]. We previously found that melatonin can alleviate hyperglycemia-induced renal inflammation by inhibiting the activation of TLR4 and the TGF-*β*1/Smad3 signaling pathway [16]. Melatonin was also reported to have a protective effect against hemin-induced platelet ferroptosis, but not against traumatic brain injury-induced ferroptosis when ferritin H was deleted in neurons [17, 18]. However, the specific mechanism of melatonin action in AKI ferroptosis has not yet been investigated.

Nuclear factor E2 related factor 2 (NRF2) is considered an important regulator of the antioxidant system. NRF2 regulates the expression of a series of signaling proteins and enzymes by targeting downstream genes; it also participates in maintaining redox homeostasis. For example, Slc7a11 (xCT), glutathione, and GPX4 are downstream target genes of NRF2 that are responsible for preventing lipid peroxidation and inhibiting ferroptosis [19]. In our study, we confirmed that melatonin treatment could ameliorate AKI by inhibiting ferroptosis *in vitro* and *in vivo* AKI models. Our research on melatonin is aimed at providing novel insights into its mechanisms and potential therapeutic benefits in AKI.

## 2. Materials and Methods

### 2.1. Animals and Drug Treatment

All animal procedures were conducted in accordance with the guidelines of the China Council on Animal Care and Use and approved by the Animal Experimentation Ethics Committee of Anhui Medical University. Male C57BL/6J mice were purchased from the Experimental Animal Center of the Anhui Medical University. Melatonin (MT) was purchased from Sigma-Aldrich (Sigma-Aldrich, St. Louis, MO) and dissolved in ethanol such that the final concentration of ethanol was 1% using physiological saline diluted. The MT solution was injected intraperitoneally at a dose of 20 mg/kg [3, 4]. MT was administered 3 days and 1 h before renal IR injury or folic acid (Sigma-Aldrich) intraperitoneal injection. Mice were intraperitoneally injected with 5 mg/kg Fer-1 (Selleck Chemicals) 30 min before renal IR injury or folic acid injection.

#### 2.1.1. Renal Ischemia/Reperfusion (IR) Model

To evaluate the protective effect of MT or Fer-1 against IR injury, male C57BL/6 mice (20–22 g, 8–10 weeks old) were randomly divided into five groups (*n* = 6): (1) Sham, (2) Sham+MT (20 mg/kg MT), (3) IR, (4) IR+MT (20 mg/kg MT), and (5) IR+Fer-1 (5 mg/kg Fer-1). MT was intraperitoneally administered 3 days and 1 h before inducing renal IR injury in mice [3]. Fer-1 was intraperitoneally administered 30 minutes before inducing renal IR injury in mice [10]. Mice were anesthetized with 50 mg/kg sodium pentobarbital by intraperitoneal injection before, and both kidneys were exposed through a dorsal incision. The renal pedicle was clamped using noninvasive forceps to induce renal ischemia for 41 min. The Sham group received dorsal incisions but not renal ischemia. All mice were euthanized 24 h after reperfusion. Blood samples were collected at the time of euthanasia, and kidneys were perfused with saline before removal.

#### 2.1.2. Folic Acid- (FA-) Induced Acute Kidney Injury (FA-AKI) Model

In the FA-AKI model, male C57BL/6 mice (25–28 g, 12–14 weeks old) were randomly divided into five groups (*n* = 6): (1) NC, (2) NC+MT (20 mg/kg), (3) FA group, (4) FA+MT (20 mg/kg), and (5) FA+Fer-1 (5 mg/kg). Male C57BL/6 mice of the same week-old or littermate mice were selected as the NC group. MT was intraperitoneally administered 3 days before and 1 d after FA injection. Fer-1 was intraperitoneally administered 30 minutes before FA injection. Mice received a single intraperitoneal injection of 250 mg/kg FA dissolved in 0.3 M sodium bicarbonate or vehicle and were euthanized 48 h later. Blood samples were collected at the time of euthanasia, and kidneys were perfused with saline before removal [10].

### 2.2. Biochemistry Analysis

Blood samples were centrifuged at 3500 × *g* for 15 min, and serum samples were collected for experiment. Hepatic and kidney function index (alanine aminotransferase (ALT), blood urea nitrogen (BUN), serum creatinine, and urine creatinine levels) were analyzed using an autoanalyzer (Hitachi 3100, Japan) at the Center for Scientific Research of Anhui Medical University. The 12-hour urine volume of ischemia-reperfusion group and 24-hour urine volume of folic acid group were collected and recorded with metabolic cage. Creatinine clearance (Ccr) was calculated using formula: Ccr = urine creatinine∗urine volume/serum creatinine. Ccr was expressed as milliliter per min.

### 2.3. Histopathological Analysis

Mouse kidneys were immediately fixed overnight with 4% paraformaldehyde after removal and then paraffin embedded. The kidney sections (4 *μ*m) were used for HE and PAS staining to assess proximal cortical renal injury. Kidney sections were blinded and randomly observed by two investigators. The HE-stained renal sections (*n* = 6–8) were evaluated tubular damage which was estimated semiquantitatively on a scale ranging from 0 to 4 points to grade the percentage of injured renal tubules: 0 corresponded to 0% injury or 0.5% to <10% injury, 1 to 10-25% injury, 2 to 26-50% injury, 3 to 51-75% injury, and 4 to 76-100% injury. The PAS-stained renal sections (*n* = 6–8) were evaluated for the proximal cortical renal damage score defined by the degree of tubular necrosis, cast formation, and tubular dilation, as shown here: 0 = normal, 1 = 10%, 2 = 10-25%, 3 = 26-50%, 4 = 51-75%, 5 = 75-95%, and 6 = greater than 96%. At least 6 areas of the kidney on each slide were randomly chosen.

### 2.4. Immunohistochemistry and Immunofluorescence Staining

Kidney sections (4 *μ*m) were first heated in 60°C, then deparaffinized in xylene, rehydrated in graded alcohol, and treated with 0.01 M sodium citrate buffer and 3% H_2_O_2_ to block endogenous peroxidase activity (immunofluorescence did not require this step). Then, 10% goat serum was added to block nonspecific antigen binding for 40 min in 37°C and in next step kidney sections were incubated with primary antibodies like anti-KIM-1 (1 : 200, Bioss), anti-CD68 (1 : 200, Proteintech), anti-TNF-*α* (1 : 200, Proteintech), anti-4-HNE (1 : 50, Abcam), and anti-Ly6G (1 : 100, Abcam) overnight at 4°C. Samples were then incubated in secondary antibodies (HRP conjugated for IHC and fluorescently labeled for IF) for 1 h at 37°C. The TdT-mediated dUTP nick-end labeling (TUNEL) staining was performed using commercial kits according to the manufacturer's instructions (Beyotime Biotechnology, Shanghai, China). DCF, DHE, and JC-1 staining was performed using commercial kits following the manufacturer's instructions (Beyotime Biotechnology, Shanghai, China). Slides were imaged using a microscope (Leica, Germany) or a confocal laser scanning microscope (Zeiss, Germany).

### 2.5. Transmission Electron Microscope (TEM)

Small pieces of the renal cortices were fixed in 2.5% glutaraldehyde, dehydrated, and embedded. The samples were then observed using a transmission electron microscope (Hitachi, Japan).

### 2.6. Detection of the Levels of SOD, MDA, GSH-Px, and Iron Content in Cell and Kidney Tissue Samples

The levels of superoxide dismutase (SOD), malondialdehyde (MDA), and glutathione peroxidase (GSH-Px) in cells and kidney tissues were analyzed with SOD assay kit, GSH-Px assay kit, and MDA assay kit (Nanjing Jiancheng Bioengineering Institute, China), according to the manufacturer's instructions. Total protein was measured using a BCA Protein Assay Kit (Beyotime Biotechnology, Shanghai, China). The iron levels in the cells and kidneys were determined using the Iron Assay Kit (Leagene Biotechnology, Beijing, China) according to the manufacturer's instructions.

### 2.7. Quantitative Real-Time PCR

Total RNA was extracted from renal tissue or cells using TRIzol reagent (Takara). Real-time PCR was performed using a CFX96 real-time PCR detection system (Bio-Rad, Hercules, CA, USA). The mRNA levels of *KIM-1*, *IL-1β*, *TNF-α*, *MCP-1*, *IL-6*, *GPX4*, *Acsl4*, *Cox-2*, *PGC-1a*, *TFAM*, and *β-actin* were quantified using SYBR Green RT-PCR. The relative gene expression was normalized by the comparative Ct (2^−*ΔΔ*Ct^) with *β-actin* gene expression. The primer sequences are listed in Table 1.

### 2.8. Western Blotting Analysis

Cells and renal tissue were lysed in cold RIPA buffer containing protease inhibitor cocktails. The total protein concentrations were quantified using a BCA protein assay kit. Equal amounts of total protein were separated in 10–15% SDS-PAGE, and samples were transferred onto nitrocellulose membranes, blocked with 5% skimmed milk in TBS/0.5% Tween (TBST) for 1 hour, washed with TBST for three times, and incubated with primary antibodies against MFN2 (1 : 500, Affinity Biosciences), UCP2 (1 : 500, Affinity Biosciences), NRF2 (1 : 500, Proteintech), Slc7a11 (1 : 1000, Abcam), GPX4 (1 : 500, Proteintech), and *β*-actin (1 : 1000, Proteintech) overnight at 4°C. The nitrocellulose membranes were washed with TBST for three times, 10 min each, and then incubated with secondary antibodies (1 : 10000, Proteintech) for 1 h at room temperature and then washed with TBST for three times again and added with the enhanced ECL Chemiluminescent Substrate Kit (Yeasen Biotechnology, Shanghai, China) in the membrane. The relative intensity of the target band was detected using the AmerSham Imager 600 image system (GE, USA). Images were quantified using ImageJ software (NIH, USA).

### 2.9. Cell Culture and Treatment

Mouse tubular epithelial cells (MTEC) were provided by Professor Huiyao Lan from the Chinese University of Hong Kong and cultured in 5% FBS-containing DMEM/F12 medium at 37°C and 5% CO_2_. In the HR group, the cells were seeded in 6-well plates for 24 h, pretreated with 1 mM melatonin or 1 *μ*M Fer-1 for 6 h, and then MTEC were subjected to hypoxia (94% N_2_, 5% CO_2_, and 1% O_2_) for 12 h, followed by reoxygenation (normoxic condition) for another 6 h. For the Era group, the cells were seeded in 6-well plates for 24 h and pretreated with 1 mM melatonin for 6 h, and then, 10 *μ*M Era was added for another 24 h.

### 2.10. MTT Assay

We used the MTT assay to detect cell viability. MTEC were grown in 96-well plates and pretreated with MT or Fer-1 for 6 h and followed by hypoxia for 12 h, reoxygenation for 6 h or Erastin for 24 h. Then, MTT (5 mg/mL) solution was added and incubated for 4 h. The absorbance was determined using a microplate reader (BIO-RAD, Thermo, USA) at a wavelength of 492 nm for optical density (OD) measurements.

### 2.11. Flow Cytometry

Apoptotic cell counts were performed in MTEC using the Annexin V–FITC/PI Apoptosis Detection Kit (Bestbio, China) according to the manufacturer's instructions, analyzed on a CytoFlex flow cytometer machine (Beckman Coulter, USA). The data were analyzed by the CytExpert software.

### 2.12. RNA-Sequencing Profile

Total RNA was extracted using the mirVana miRNA Isolation Kit (Ambion) following the manufacturer's protocol. RNA integrity was evaluated using an Agilent 2100 Bioanalyzer (Agilent Technologies, USA). Samples with an RNA integrity number (RIN) ≥ 7 were used for subsequent analysis. Transcriptome sequencing and analysis were conducted by OE Biotech Co., Ltd. (Shanghai, China). In the fragments per kilobase of transcript per million mapped reads, i.e., FPKM value of each gene was calculated using cufflinks, and the read counts of each gene were obtained using htseq-count. Differentially expressed genes (DEGs) were identified using the DESeq (2012) R package functions estimateSizeFactors and nbinomTest. *p* values < 0.05, and fold change > 1.5 were used as thresholds for evaluating significant differential expression. Hierarchical cluster analysis of DEGs was performed to explore the gene expression patterns. Gene ontology (GO) and KEGG pathway enrichment analyses of DEGs were performed based on their hypergeometric distribution using R.

### 2.13. Chromatin Immunoprecipitation (ChIP) Assay

To confirm that NRF2 directly binds to the promoter of Slc7a11, ChIP assays were performed using the Enzymatic ChIP kit (Cell Signaling Technology). MTEC were incubated with or without melatonin for 24 h and fixed in 1% formaldehyde to cross-link DNA and proteins at room temperature for 10 min, followed by the addition of 10 × glycine to stop cross-linking. Next, the MTEC were washed twice with cold PBS and 2 ml cold PBS+10 *μ*l protease inhibitor cocktail (PIC). After cell lysis, nuclei were isolated by centrifugation, and the cross-linked chromatin was sheared into 150–900 bp fragments by ultrasonication. Chromatin was immunoprecipitated with an anti-NRF2 antibody or a negative control IgG antibody at 4°C overnight. DNA samples were purified using magnetic beads and amplified by ChIP-qPCR using the following primers: F, 5′-TTGAGCAACCCACAGGCTAC-3 and R, 5′-GCATCAGCCACATGAGAAAA-3′. The PCR products were added to a 1% agarose gel with GelRed nucleic acid gel stain, and images were captured using the Tanon gel documentation system.

### 2.14. Transfection

**Figure 1:**
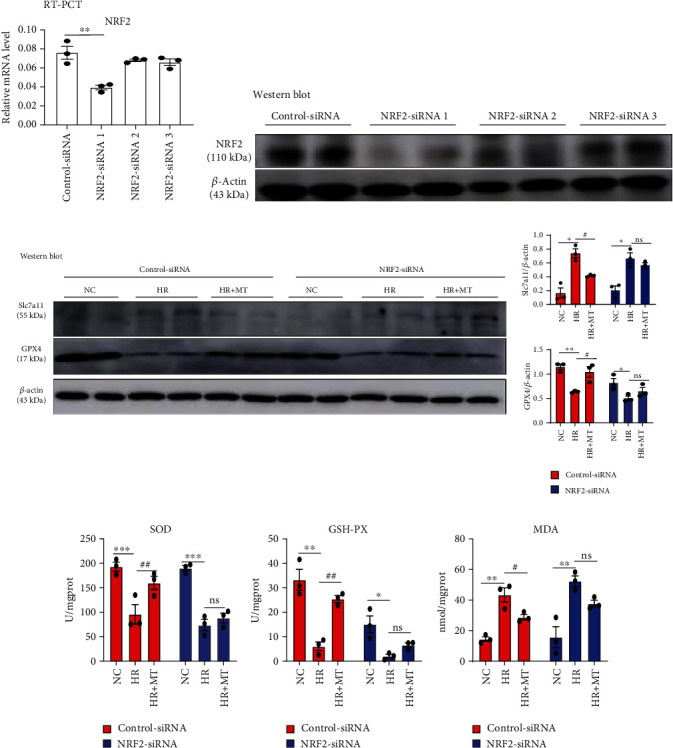
Melatonin failed to alleviate the HR-induced ferroptosis in NRF2-silenced MTEC. (a) Real-time PCR and (b) western blot analysis of the expression of NRF2 in MTEC after transfection with control siRNA or three alternative *NRF2* siRNAs (NRF2 siRNAs 1, 2, and 3), respectively. (c) Western blotting showing the expression of Slc7a11 and GPX4 proteins in control-siRNA and *NRF2*-siRNA cells treated with HR and 1 mM melatonin. (d–f) The levels of SOD, GSH-Px, and MDA in control-siRNA and *NRF2*-siRNA cells treated with HR and 1 mM melatonin were measured. Data represent the mean ± SEM of three to four independent experiments. ^∗^*p* < 0.05,  ^∗∗^*p* < 0.01, and^∗∗∗^*p* < 0.001 compared with the NC group. ^#^*p* < 0.05,  ^##^*p* < 0.01, and^###^*p* < 0.001 compared with the HR-treated group.

The MTEC were seeded into 6-well plates and transfected with *NRF2* or control siRNA (Hanbio Tec, Shanghai, China) using Lipofectamine 2000 reagent (Invitrogen, Carlsbad, CA, USA). The target sequences for the preparation of siRNAs of mouse *NRF2* are listed in Table 2. The siRNA and Lipofectamine 2000 were gently mixed, incubated at room temperature for 20 min, and transferred into cells. After 6 h incubation, the cells were washed twice with PBS and cultured in 5% FBS-containing DMEM/F12. The effect of siRNA-induced gene silencing was verified using real-time quantitative PCR and western blotting (Figures 1(a) and 1(b)).

### 2.15. Statistical Analyses

All data were shown as the mean ± SEM, and the one-way analysis of variance (ANOVA) was used for data analysis, followed by Tukey's post hoc tests using GraphPad Prism 8 software.

## 3. Results

### 3.1. Melatonin Inhibits IR-Induced Acute Kidney Injury in Mice

**Figure 2:**
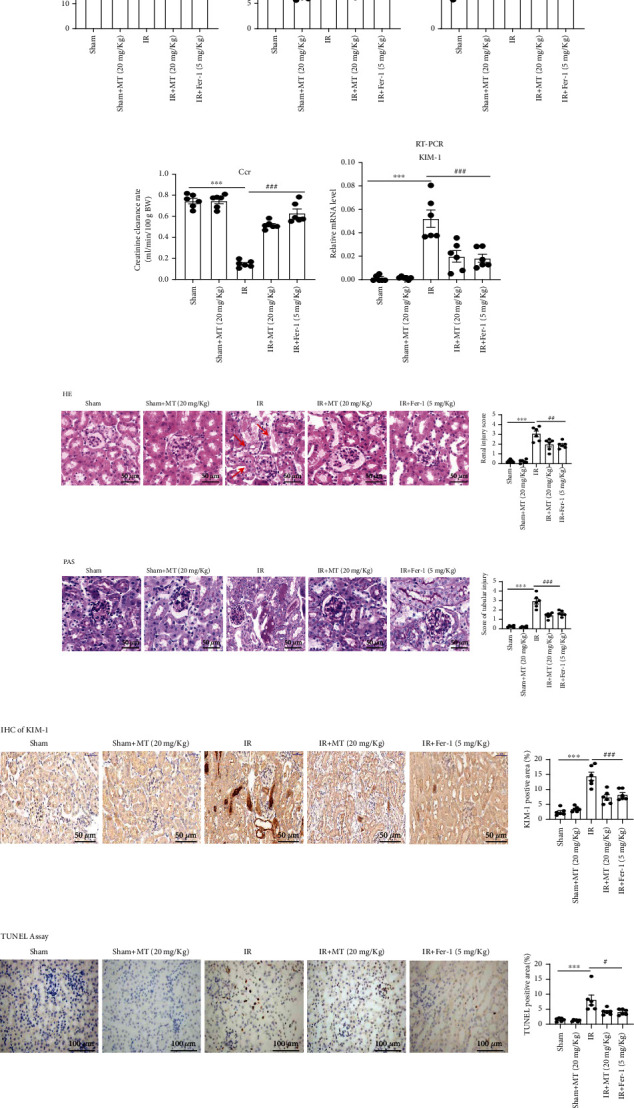
Melatonin treatment alleviates IR-induced acute kidney injury in mice. (a) Serum ALT. (b–d) BUN, serum creatinine (Scr), and Ccr level; the result shows that MT or Fer-1 could alleviate the kidney injury in IR-AKI. (f, g) Representative image of HE and PAS revealed that renal tubular injury induced by IR was partly reversed by MT and Fer-1 treatment, and renal tubular damage was scored semiquantitatively (right panel). (e) Real-time PCR and (f) immunohistochemistry were used to measure the expression of KIM-1 in kidney tissues. (i) TUNEL staining and scores. Data represent the mean ± SEM of six mice in each group. ^∗^*p* < 0.05,  ^∗∗^*p* < 0.01, and^∗∗∗^*p* < 0.001 compared to the Sham and Sham+MT (20 mg/kg) groups. ^#^*p* < 0.05,  ^##^*p* < 0.01, and^###^*p* < 0.001 compared to the IR-treated group. Scale bar = 50-100 *μ*m.

Previous reports have shown that ferroptosis plays an important role in AKI [5–10]. To demonstrate the role of melatonin in ferroptosis, a bilateral renal IR model was established in mice. Melatonin itself had no effect on hepatic function (Figure 2(a)), BUN, or serum creatinine levels in normal mice. Compared with the Sham and Sham+MT groups, IR-induced increases in BUN and serum creatinine levels and decreases in Ccr were partly restored with melatonin and Fer-1 treatments (Figures 2(b)–2(d)). Tubular injury was assessed by HE (Figure 2(f)) and PAS staining (Figure 2(g)) in kidney sections, which showed that melatonin and Fer-1 treatments reduced histological injury when compared with the IR group. Similarly, IR-induced mRNA and protein expression of KIM-1, which is a molecular marker for tubular injury, were also suppressed by melatonin and Fer-1 treatment (Figures 2(e) and 2(h)). In addition, terminal deoxynucleotidyl transferase-mediated digoxigenin deoxyuridine nick-end labeling (TUNEL) analysis showed that melatonin and Fer-1 treatments attenuated IR-induced cell death when compare with the IR group (Figure 2(i)).

### 3.2. Melatonin Ameliorates IR-Induced Inflammatory Responses

**Figure 3:**
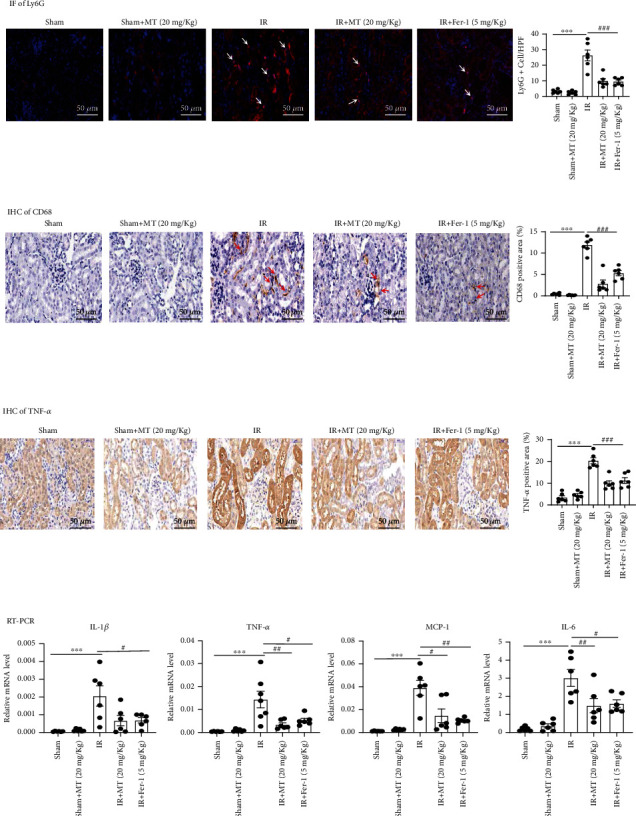
Melatonin treatment attenuates the IR-induced renal inflammatory response. (a–c) Melatonin alleviated neutrophil and macrophages recruitment, and then secretion of TNF-*α* in kidney sections labeled with antibodies against Ly6G, CD68, and TNF-*α* and score. (d) The mRNA expression levels of *IL-1β*, *TNF-α*, *MCP-1*, and *IL-6* in the kidneys was evaluated. Data are presented as the mean ± SEM for six mice in each group. ^∗^*p* < 0.05,  ^∗∗^*p* < 0.01, and^∗∗∗^*p* < 0.001 compared to the Sham and Sham+MT (20 mg/kg) groups. ^#^*p* < 0.05,  ^##^*p* < 0.01, and^###^*p* < 0.001 compared to the IR-treated group. Scale bar = 50 *μ*m.

The inflammatory response plays a key role in the pathogenesis of acute kidney injury [20–22]. Recent studies have shown that local inflammation caused by ferroptosis can exacerbate kidney damage [10]. Immunofluorescence staining of neutrophils (Ly6G), immunohistochemistry of macrophages (CD68), and inflammatory cytokines (TNF-*α*) were performed in kidney sections to test inflammatory responses. Inflammatory cell infiltration and inflammatory factor secretion were reduced by melatonin or Fer-1 treatment in the kidney when compared with that observed in the IR group (Figures 3(a)–3(c)). We further assessed the expression of the proinflammatory cytokines *IL-1β*, *TNF-α*, *MCP-1*, and *IL-6* to evaluate the effect of melatonin on inflammation associated with IR. We found that the expression of above proinflammatory cytokines was inhibited with melatonin or Fer-1 treatment when compared with the IR group (Figure 3(d)).

### 3.3. Melatonin Improves Mitochondrial Function after Renal IR

**Figure 4:**
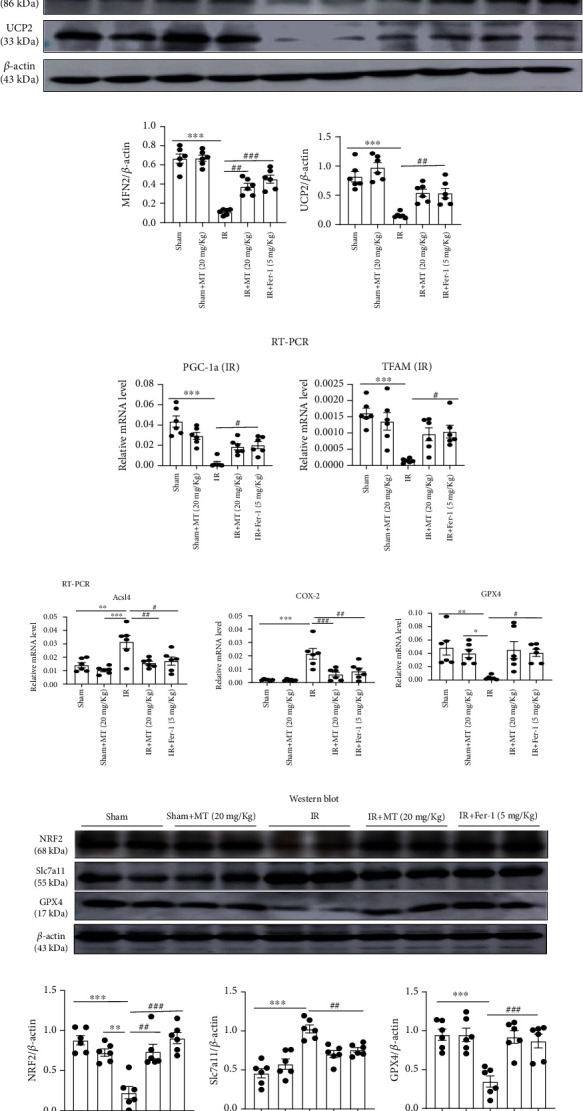
Melatonin improves mitochondrial function and inhibits ferroptosis in the IR-induced acute kidney injury. (a) Renal tissues were imaged using transmission electron microscopy (TEM). Red arrows indicate mitochondrial cristae disappearance and outer membrane rupture. Scale bar = 200 nm. (b) Western blot analysis of mitochondrial proteins and their relative protein levels of UCP2 (c) and MFN2 (d). (e) Renal relative PGC-1a and TFAM levels were evaluated by RT-PCR. (f) Quantification of mRNA levels of *Acsl4*, *Cox-2*, and *GPX4* kidney tissue by real-time PCR. (g) Western blots of NRF2, Slc7a11, and GPX4 proteins and their relative protein levels of NRF2 (h), Slc7a11 (i), and GPX4 (j). Data represent the mean ± SEM of six mice in each group. ^∗^*p* < 0.05,  ^∗∗^*p* < 0.01, and^∗∗∗^*p* < 0.001 compared to the Sham and Sham+MT (20 mg/kg) groups. ^#^*p* < 0.05,  ^##^*p* < 0.01, and^###^*p* < 0.001 compared to the IR-treated group.

The mitochondria morphological features of ferroptosis were small mitochondria with condensed mitochondrial membrane density, reduction, or vanishing of mitochondria crista, as well as outer mitochondrial membrane rupture. We observed the decrease of mitochondrial crista and outer mitochondrial membrane rupture in renal tubular epithelial cells of the renal ischemia-reperfusion group under transmission electron microscopy. And melatonin and Fer-1 pretreatment alleviated these effects of IR (Figure 4(a)). Mitochondria are the energetic, metabolic, redox, and information signaling centers of the cell. Substrate pressure, mitochondrial network dynamics, and cristae morphology state are integrated by the proton motive force or its potential component *ΔΨ*m, which are attenuated by proton backflux into the matrix, termed uncoupling. The mitochondrial uncoupling proteins (UCP) play an eminent role in the regulation of each of the above aspects, being involved in numerous physiological events including redox signaling. The mitochondrial uncoupling proteins (UCP) play an eminent role in the regulation of each of the above aspects, being involved in numerous physiological events including redox signaling. And the mitochondrial biogenesis proteins (PGC1*α*/Tfam in mRNA level) and fusion protein (Mfn-2 in protein level) were essential for mitochondrial biogenesis [23]. Melatonin and Fer-1 treatment increased the expression of UCP2 and MFN2 in renal tissue of IR mice (Figures 4(b)–4(d)). In addition, the mRNA expression of markers for mitochondrial biogenesis functions [23], such *PGC-1α* and *TFAM*, was restored after melatonin and Fer-1 treatment in renal tissue of IR mice (Figure 4(e)).

### 3.4. Melatonin Alleviates IR-Induced Ferroptosis

**Figure 5:**
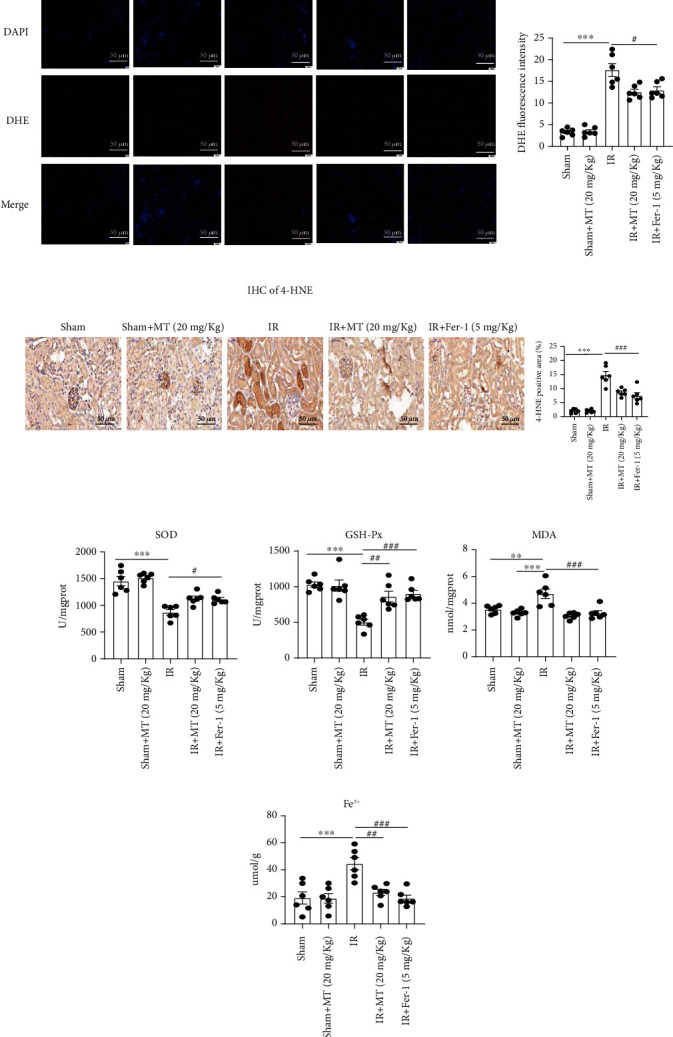
Melatonin reduces IR-induced ferroptosis in the AKI mouse model. (a) The fluorescent probe DHE (red) indicating ROS in kidneys sections from IR-induced mice. (b, e) Lipid peroxidation levels were measured by 4-HNE and MDA in kidney tissue. (c, d) SOD and GSH-Px levels in kidney tissue after IR-AKI. (f) Fe^2+^ concentration in kidney tissue after IR-AKI. Data are presented as the mean ± SEM for six mice in each group. ^∗^*p* < 0.05,  ^∗∗^*p* < 0.01, and^∗∗∗^*p* < 0.001 compared to the Sham and Sham+MT (20 mg/g) groups. ^#^*p* < 0.05,  ^##^*p* < 0.01, and^###^*p* < 0.001 compared to the IR-treated group. Scale bar = 50 *μ*m.

Increasing evidence indicates that ferroptosis plays a major role in the cell death induced by acute kidney injury. As a biomarker of ferroptosis, we analyzed xCT/Slc7a11, a cystine/glutamate antiporter; Acsl4 and Cox-2, two key enzymes in ferroptosis-sensitive phospholipid biosynthesis; and GPX4, a well-known ferroptosis regulator. We found that treatment of melatonin or Fer-1 inhibited IR-induced upregulation of Slc7a11 (Figure 4(g)), Acsl4 (Figure 4(f)), and Cox2 (Figure 4(f)) and restored IR-induced downregulation of GPX4 and NRF2 (Figures 4(f) and 4(g)). Reduced glutathione levels can cause the accumulation of lipid hydroperoxides and thus trigger ferroptosis. We found that both melatonin and Fer-1 inhibited IR-induced increase in renal ROS formation (DHE staining) (Figure 5(a)) and improved IR-induced downregulation of SOD and GSH-Px levels (Figures 5(c) and 5(d)). Furthermore, the upregulation of the ferroptosis end-products, MDA and 4-hydroxynonenal (4-HNE), was ameliorated by melatonin and Fer-1 treatment (Figures 5(b) and 5(e)). Iron accumulation can also contribute to ferroptosis, and the level of Fe^2+^ can reflect iron overload. High levels of Fe^2+^ can produce highly active hydroxyl radicals through the Fenton reaction, leading to the accumulation of lipid hydroperoxides. We found that Fe^2+^ levels were upregulated in the IR-induced AKI model compared with the Sham group and that melatonin and Fer-1 treatment could partially inhibit the upregulation of Fe^2+^ in the AKI model (Figure 5(f)). Collectively, our results suggest that IR-AKI alters the expression of ferroptosis-related genes and proteins, as well as several known ferroptosis biomarkers. However, treatment of IR-AKI with melatonin and Fer-1 was effective in rescuing IR-induced ferroptosis.

### 3.5. Melatonin Suppresses Cell Death, Mitochondrial Dysfunction, and ROS Production

**Figure 6:**
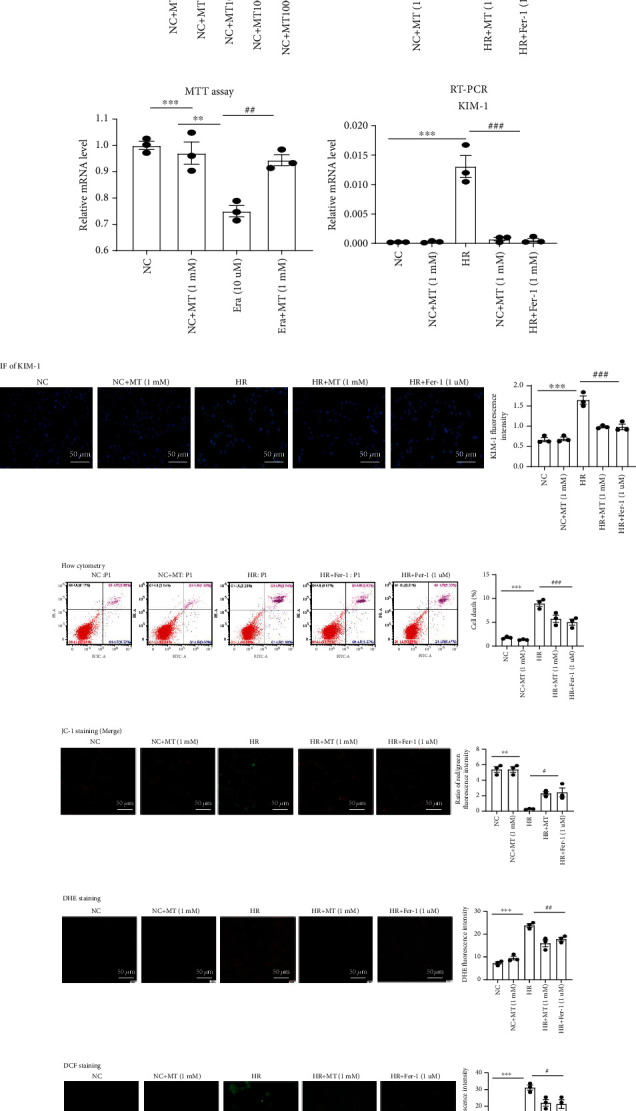
Melatonin reduces the HR-induced cell death, mitochondrial dysfunction, and ROS production. (a) Cells were treated with melatonin (10–10,000 *μ*M) for 24 h and then assessed using the MTT assay. ^∗^*p* < 0.05 vs. the NC group. (b, c) The MTT assay was used to evaluate the effects of melatonin and Fer-1 on HR- or Era-induced cytotoxicity. MTEC were pretreated with or without 1 mM melatonin or 1 *μ*M Fer-1 for 6 h and then treated with HR or 10 *μ*M Era. (d, e) Real-time PCR and immunofluorescence analysis of KIM-1 in MTEC. (f) The death of MTECs induced by HR was analyzed by flow cytometry. The results showed that melatonin and Fer-1 could reduce MTECs death induced by HR. (g) mitochondrial membrane potential (MMP) measured using JC-1. In the normal MTEC, most JC-1 formed J-aggregates and showed red fluorescence at 525/590 nm; in damaged MTEC, most JC-1 became monomer in mitochondrial matrix because *Δψ*m decreased and showed green fluorescence at 490/530 nm. Under an inverted fluorescence microscope, we merged both the JC-1 pictures in 525/590 nm and 490/530 nm in the same field to acquire the pictures of *Δψ*m. The NC or NC+MT (1 mM) MTEC mainly showed red fluorescence; in the HR group, green fluorescence increased and red fluorescence lessened, which was reversed in the MT (1 mM) and Fer-1 (1 *μ*M) groups. (h, i) DHE and DCF staining were used to measure ROS production in MTEC. Results showed that ROS were upregulated induced by HR, and melatonin and Fer-1 could reduce the upregulation of ROS induced by HR. Data represent the mean ± SEM of 3–4 independent experiments. ^∗^*p* < 0.05,  ^∗∗^*p* < 0.01, and^∗∗∗^*p* < 0.001 compared to the NC and NC+MT (1 mM) groups. ^#^*p* < 0.05,  ^##^*p* < 0.01, and^###^*p* < 0.001 compared with the HR- or Era-treated group. Scale bars = 20-50 *μ*m.

To determine whether melatonin influences MTEC viability, cells were treated with different melatonin concentrations for 24 h. While 10–1000 *μ*M melatonin had no detectable cytotoxic effect on cell viability, 10,000 *μ*M melatonin had an observable cytotoxic effect on the viability of MTEC (*p* < 0.05, Figure 6(a)). We then induced MTEC cytotoxicity with HR and Era and found that both MT and Fer-1 could ameliorate HR-induced or Era-induced cytotoxicity (Figures 6(b) and 6(c)). Next, we evaluated the changes of KIM-1 levels under HR conditions by real-time PCR and immunofluorescence; the results showed that melatonin and Fer-1 treatment could reduce KIM-1 expression compare with the HR group (Figures 6(d) and 6(e)). In addition, flow cytometry analysis Annexin V–FITC/PI staining showed that melatonin and Fer-1 alleviated HR-induced programmed cell death (Figure 6(f)). Next, it was investigated whether melatonin and Fer-1 protected cells from HR-induced mitochondrial dysfunction and ROS production. As shown in Figure 6(g), mitochondrial membrane potential (MMP) was significantly reduced in HR-treated cells but enhanced by melatonin and Fer-1 treatment. Similarly, HR significantly increased ROS production, which was alleviated by melatonin and Fer-1 treatment (Figures 6(h) and 6(i)).

### 3.6. Melatonin Prevents Ferroptosis in HR/Era-Induced MTEC

**Figure 7:**
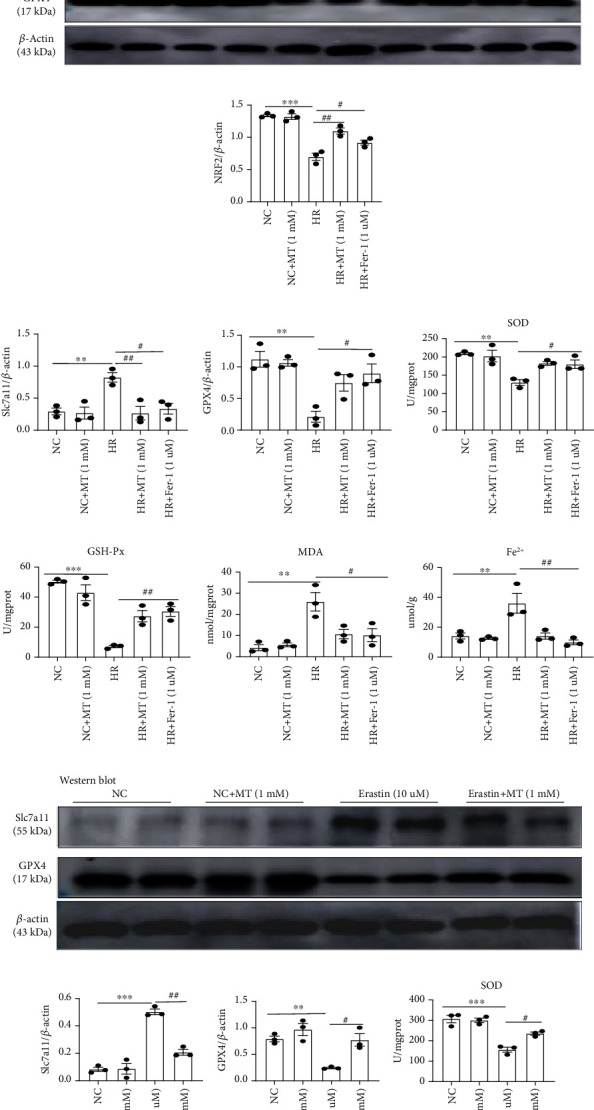
Melatonin inhibits HR- or Era-induced ferroptosis *in vitro*. (a) Quantification of *Acsl4*, *Cox-2*, and *GPX4* mRNA levels by real-time PCR in the HR group. (b–e) Western blotting of NRF2, Slc7a11, and GPX4 in HR-treated MTEC. (f–i) The levels of SOD, GSH-Px, MDA, and Fe^2+^ were measured in HR-treated MTEC, respectively. (j–l) Quantitative data analysis of western blots for Slc7a11 and GPX4 in the Era-treated group. (m–o) The levels of SOD, GSH-Px, and MDA were measured in Era-treated MTEC. Data represent the mean ± SEM of 3–4 independent experiments. ^∗^*p* < 0.05,  ^∗∗^*p* < 0.01, and^∗∗∗^*p* < 0.001 compared with the NC and NC+MT (1 mM) groups. ^#^*p* < 0.05,  ^##^*p* < 0.01, and^###^*p* < 0.001 compared with the HR- or Era-treated group.

To further determine whether melatonin protects renal tubular cells against ferroptosis, MTEC were simulated with hypoxia, reoxygenation, or Era to induce ferroptosis. As shown in Figures 7(a)–7(e), melatonin and Fer-1 administration inhibited HR-induced upregulation of Slc7a11, Acsl4, and Cox-2 mRNA and protein expression. Likewise, the expression of GPX4 decreased after HR induction but was restored by melatonin and Fer-1 treatments. We also measured glutathione metabolism, lipid peroxidation, and iron content in the HR-induced cell injury model and found that melatonin and Fer-1 inhibited HR-induced decreases in SOD and GSH-Px, as well as the upregulation of MDA and Fe^2+^ levels (Figures 7(f)–7(i)). Similarly, our results showed that melatonin mitigated ferroptosis induced by Era in MTEC (Figures 7(j)–7(o)). Taken together, our data indicate that melatonin inhibited HR- and Era-induced ferroptosis in MTEC.

### 3.7. Melatonin Prevents Ferroptosis in MTEC through NRF2/Slc7a11 Axis

**Figure 8:**
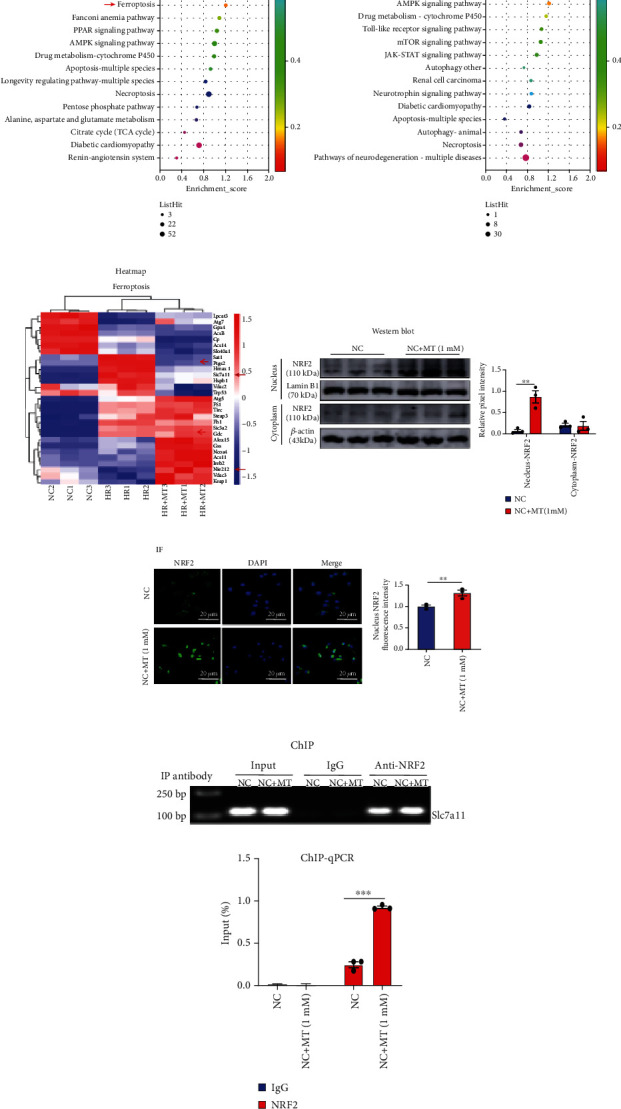
Melatonin upregulates the expression of *NRF2* and enhanced NRF2 binding to the *Slc7a11* promoter to inhibit ferroptosis. (a) KEGG pathway classification and enrichment of differentially expressed genes in the HR versus NC groups; HR-induced cell death included apoptosis, necroptosis, autophagy, and ferroptosis. (b) The results of the KEGG pathway enrichment analysis of differentially expressed genes (DEGs) showed that glutathione metabolism and ferroptosis (red arrow), more than apoptosis, autophagy, and necroptosis, were among the highly enriched pathways in the HR+MT versus HR groups. (c) Heat map of the KEGG pathway clustering of genes of the ferroptosis pathway in the NC, HR, and HR+MT groups; the expression of Slc7a11 was significantly increased after HR treatment and was remarkably restored by MT therapy. (d, e) MTEC were incubated with or without melatonin for 24 h. Western blotting and immunofluorescence (confocal laser scanning microscope) showing that melatonin promoted NRF2 nuclear translocation. Scale bar = 20 *μ*m. (f, g) ChIP assays and ChIP-qPCR analysis of NRF2 binding to *Slc7a11* in MTEC treated with or without melatonin. Data represent the mean ± SEM of 3–4 independent experiments. ^∗^*p* < 0.05,  ^∗∗^*p* < 0.01, and^∗∗∗^*p* < 0.001, compared with the NC group.

Next, we performed RNA-sequence analysis to elucidate the molecular mechanisms by which melatonin protects renal tubular cells against ferroptosis. KEGG pathway enrichment analysis detected enriched genes in the HR group when compared with the NC group, including apoptosis, necroptosis, autophagy, and ferroptosis (Figure 8(a)). However, compared with the HR group and the HR+MT group, glutathione metabolism and ferroptosis pathways had higher enrichment scores than apoptosis, necroptosis, and autophagy, indicating the main pathways of melatonin in the treatment of HR were glutathione metabolism and ferroptosis pathway (Figure 8(b)). A heat map of ferroptosis-related genes showed that the expression of *Slc7a11* was significantly upregulated and the expression of *NRF2* was significantly downregulated after HR treatment; nevertheless, the expression of both *Slc7a11* and *NRF2* was restored by melatonin therapy after HR treatment (Figure 8(c)). Similarly, in the HR- or IR-induced AKI model, NRF2 protein levels were efficiently increased after melatonin and Fer-1 treatments (Figures 5(d) and 7(b)).

NRF2 regulates the expression of a series of signal proteins and enzymes through downstream target genes to maintain redox homeostasis. Like Slc7a11, glutathione and GPX4 are downstream target genes of NRF2 that are responsible for preventing lipid peroxidation and thus, inhibiting ferroptosis [19, 24]. Under stressed conditions, NRF2 translocates into the nucleus and activates the transcription of cytoprotective genes. To investigate whether melatonin promotes NRF2 translocation into the nucleus, we measured NRF2 nuclear protein expression and nuclear translocation using western blotting and immunofluorescence. As shown in Figures 8(d) and 8(e), melatonin enhanced NRF2 nuclear protein expression and nuclear translocation compared to the NC group. Next, we performed a chromatin immunoprecipitation (ChIP) assay to verify that melatonin promoted NRF2 binding to the Slc7a11 promoter to induce its transcriptional expression. The chromatin of these cells was precipitated using an anti-NRF2 antibody, and oligonucleotide primers were used to amplify the *Slc7a11* promoter region from DNA isolated from the immunoprecipitation complexes of control and melatonin-treated cells. The binding of NRF2 to the *Slc7a11* promoter region was significantly enhanced in melatonin-treated cells when compared with the NC group, indicating enhanced promoter activity (Figures 8(f) and 8(g)).

Lastly, we determined whether melatonin could mediate the protective effect of the NRF2/Slc7a11 axis to inhibit ferroptosis. In MTEC in which NRF2 was knocked down, the protein expression of Slc7a11 and GPX4 was not further protected by melatonin when compared with control-siRNA cells in HR-treated MTEC (Figure 1(c)). We observed that cells transfected with NRF2-siRNA significantly attenuated the antiferroptosis effects of melatonin, as evidenced by a decrease in SOD and GSH-Px, accompanied by a significant increase in MDA levels (Figures 1(d)–1(f)). Together, our results suggest that the protective effects of melatonin against HR-induced ferroptosis in MTEC required functional NRF2.

### 3.8. Melatonin Ameliorates Ferroptosis in FA-AKI

**Figure 9:**
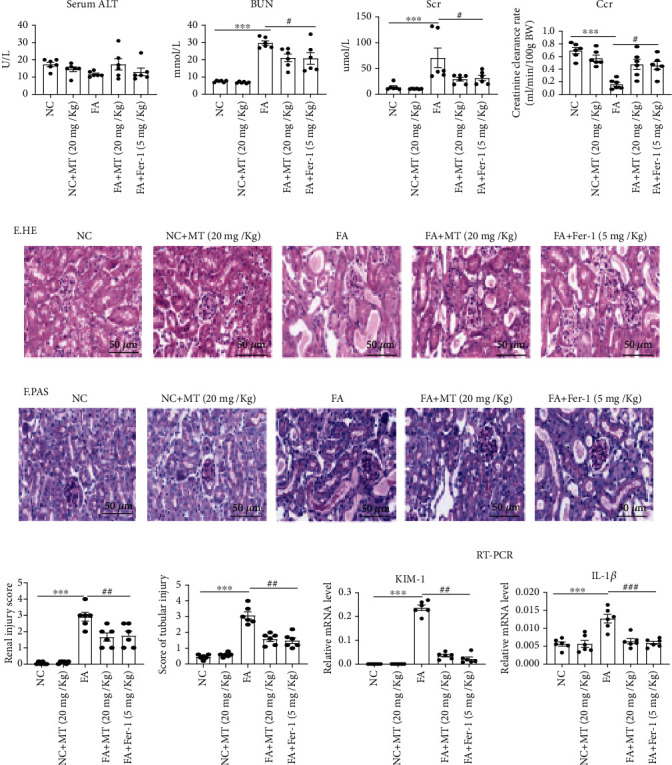
Melatonin prevents FA-induced acute kidney injury in mice. (a) Serum ALT. (b–d) BUN, serum creatinine (Scr), and Ccr level; the result shows that MT or Fer-1 could alleviate the kidney injury in FA-AKI. (e–h) Representative image of HE and PAS and tubular damage was scored semiquantitatively. (i) Real-time PCR of *KIM-1* and *IL-1β* expression in FA-AKI model. Data represent the mean ± SEM for 6 mice in each group. ^∗^*p* < 0.05,  ^∗∗^*p* < 0.01, and^∗∗∗^*p* < 0.001 compared with the NC and NC+MT (20 mg/kg) groups. ^#^*p* < 0.05,  ^##^*p* < 0.01, and^###^*p* < 0.001 compared with the folic acid- (FA-) treated group. Scale bar = 50 *μ*m.

**Figure 10:**
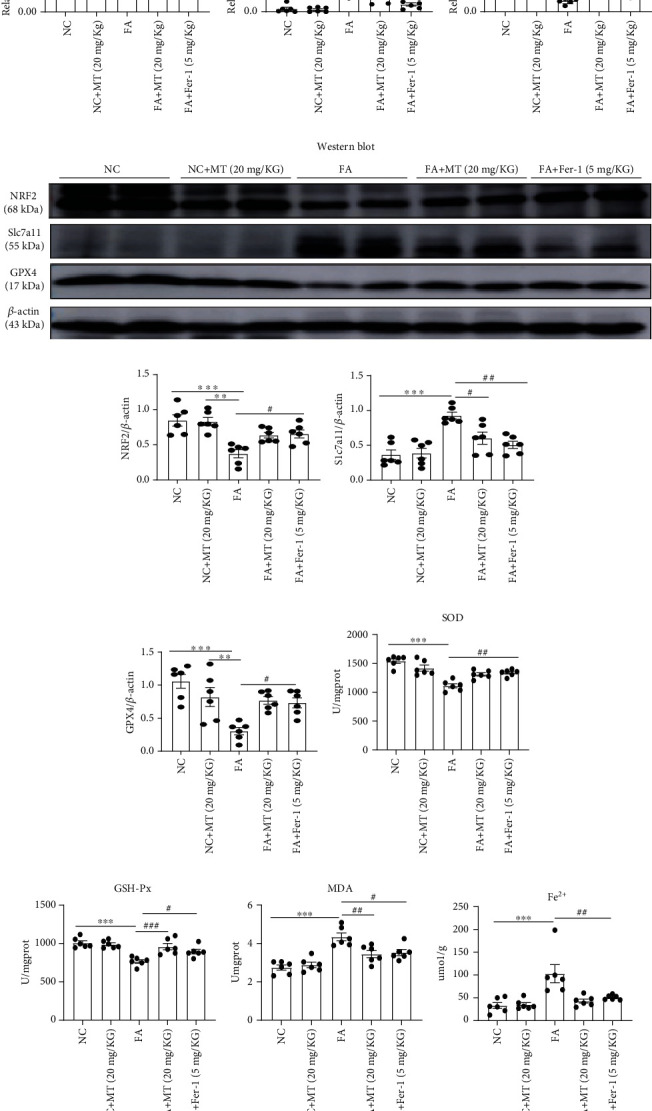
Melatonin inhibited FA-induced ferroptosis in mice. (a) Quantification of *Acsl4*, *Cox-2*, and *GPX4* mRNA levels in kidney tissue by real-time PCR; the results showed that treatment of melatonin or Fer-1 inhibited FA-induced upregulation of *Acsl4* and *Cox2* mRNA levels and restored FA-induced downregulation of *GPX4* mRNA levels. (b–e) Quantitative data analysis of western blots of NRF2, Slc7a11, and GPX4 protein expression; the results showed that treatment of melatonin or Fer-1 inhibited FA-induced upregulation of Slc7a11 protein and restored FA-induced downregulation of NRF2 and GPX4 proteins. Thank you for your revision (f–i) Levels of SOD, GSH-Px, MDA, and Fe^2+^ were measured in the kidney tissue of FA-AKI mice; the results showed MT or Fer-1 reduced the accumulation of lipid peroxidation in kidney after folic acid injury. Data represent the mean ± SEM for 6 mice in each group. ^∗^*p* < 0.05,  ^∗∗^*p* < 0.01, and^∗∗∗^*p* < 0.001 compared with the NC and NC+MT (20 mg/kg) groups. ^#^*p* < 0.05,  ^##^*p* < 0.01, and^###^*p* < 0.001 compared with the FA-treated group.

Folic acid-induced AKI is a classic model characterized by tubular cell death, tubulointerstitial inflammatory cell infiltration, and tubular regeneration. Studies have shown that ferroptosis is critical in the pathogenesis of FA-AKI [10]. Therefore, we investigated the effect of melatonin on a folic acid-induced AKI mouse model. We found that BUN and serum creatinine were significantly increased and Ccr significantly decreased after folic acid-induced AKI but partly restored after melatonin and Fer-1 treatment (Figures 9(a)–9(d)). Tubular injury was evaluated with HE and PAS staining in kidney sections, and it was showed that melatonin and Fer-1 significantly reduced histological injury (Figures 9(e)–9(h)). We also found that *KIM-1* and *IL-1β* upregulation was alleviated by the treatment with melatonin and Fer-1 in FA-AKI mice (Figure 9(i)). Induction of ferroptosis, including expression of Slc7a11 protein, *Acsl4* and *Cox-2* gene expression, and the levels of MDA and Fe^2+^, significantly increased 48 h after folic acid treatment, but it was partly reversed with melatonin and Fer-1. Likewise, melatonin and Fer-1 inhibited the decrease in NFR2, GPX4, SOD, and GSH-Px levels in kidney tissue 48 h after folic acid treatment (Figures 10(a)–10(i)). Thus, our data demonstrate that melatonin protected against ferroptosis caused by folic acid-induced AKI.

## 4. Discussion

AKI is mainly caused by multiple clinical factors, including ischemia-reperfusion injury, nephrotoxic drugs, sepsis, and urinary tract obstruction. AKI can be used as a marker of disease severity because it has high mortality and a poor outcome, although it is easily overlooked in clinical settings [1, 20]. There is still a lack of effective drugs for the treatment of AKI and therefore, understanding the pathophysiology of AKI is a cornerstone for exploring novel diagnostic and therapeutic strategies. In the past few decades, studies have focused on different pathways of programmed death to understand the pathogenesis of acute kidney injury, including necroptosis, autophagy, and apoptosis. Recently, some novel mechanisms have been found involved in AKI. Chen et al. found that crosstalk between connexin 32 and mitochondrial apoptotic signaling pathway plays a pivotal role in renal ischemia reperfusion-induced AKI and lipoxin A4 restores septic renal function via blocking crosstalk between inflammation and premature senescence [25, 26]. Yuan et al. reported that gap junction composed of Cx43 inhibition attenuated RIP1 and MLKL expression via mediating the content of ROS and then prevented acute kidney injury following liver transplantation [27]. However, increasing evidence suggests that ferroptosis plays an important role in ischemia-reperfusion injury, rhabdomyolysis, and folic acid-induced acute kidney injury. Zhao et al. performed scRNA-seq in an ischemia-reperfusion-induced AKI model and observed that ferroptosis-associated genes were more highly expressed than other pathway genes, indicating that ferroptosis is an important part of AKI [6]. Guerrero-Hue et al. reported that curcumin ameliorates rhabdomyolysis-induced renal injury by reducing ferroptosis-mediated cell death [28], and Martin-Sanchez et al. demonstrated that ferroptosis plays a prominent role in nephrotoxic folic acid-induced AKI [10]. Hu et al. also reported that inhibition of ferroptosis by VDR activation attenuated cisplatin-induced AKI [8]. Similarly, Chen et al. showed that legumain promotes tubular ferroptosis by facilitating chaperone-mediated autophagy of GPX4 in AKI [29]. Collectively, these studies suggest that ferroptosis is involved in the pathogenesis of AKI [30]. In our study, RNA-seq analysis showed that melatonin might alleviate AKI by improving glutathione metabolism and inhibiting ferroptosis through NRF2, and it was also confirmed *in vivo* that melatonin and Fer-1 (specific inhibitor of ferroptosis) could alleviate AKI induced by ischemia-reperfusion and folic acid by inhibiting ferroptosis.

Ferroptosis was first proposed by Scott et al. in 2012 and is completely different from other types of programmed cell death such as apoptosis, necroptosis and autophagy, and pyroptosis at morphological, biochemical, and genetic levels [11]. Ferroptosis is typically characterized by iron accumulation, lipid peroxidation, and mitochondrial dysfunction, and the assessment of lipid peroxidation and iron accumulation is necessary to determine whether ferroptosis has occurred [11, 31, 32]. Importantly, system X_c_^−^ (xCT), a cystine/glutamate antiporter, is responsible for the generation of intracellular GSH against oxidative stress, and its inhibition by erastin or sorafenib depletes GSH levels and decreases the activity of glutathione peroxidase 4 (GPX4), increasing lipid peroxidation and triggering ferroptosis [33, 34]. Friedmann Angeli et al. reported that inactivation of GPX4, which is a ferroptosis regulator, can trigger acute renal failure in mice [13]. Iron transport and storage are related to ferroptosis sensitivity. Zarjou et al. demonstrated that proximal tubule H-ferritin mediates iron trafficking during acute kidney injury [35], and therefore, lipid peroxidation and iron homeostasis are important for in the pathophysiology of AKI.

Melatonin is a pineal hormone that has been known to be an effective antioxidant with beneficial effects on many pathological conditions [36]. Melatonin has been reported to promote renal regeneration in FA-induced AKI by inhibiting the nucleocytoplasmic translocation of HMGB1 in tubular epithelial cells [37]. In addition, melatonin has a significant effect on apoptosis and necroptosis in cisplatin-induced AKI [3]. Also, in a retrospective cohort study of hospitalized patients, melatonin use was associated with a significant reduction in vancomycin-related acute kidney injury [38]. In the study of chronic kidney disease (CKD), melatonin can be used for targeting the intrarenal renin-angiotensin system, delaying kidney injury, and improving sleep disorders and blood pressure rhythm changes in CKD [39, 40]. And in the study of polycystic kidney disease, melatonin by lowering oxidative damage to the renal tubular cells may similarly improve cysts in both mammalian and *Drosophila* renal tubules [41]. We previously showed that melatonin could ameliorate hyperglycemia-induced renal inflammation by inhibiting the activation of TLR4 and the TGF-*β*1/Smad3 signaling pathway and, more recently [16], melatonin was reported to have a protective effect against hemin-induced platelet ferroptosis and traumatic brain injury-induced ferroptosis [17, 18]. However, the mechanism by which melatonin exerts its renoprotective effect on ferroptosis in AKI requires further research. To our knowledge, this is the first study in an AKI model to elucidate the protective role of melatonin against ferroptosis. Here, we confirmed that the downregulation of SOD, GSH, and GPX4 levels; the upregulation of Slc7a11, Acsl4, Cox2, 4-HNE, MDA, and Fe^2+^ levels; and kidney dysfunction all occur in the AKI mouse model. We observed that melatonin and Fer-1 reversed these effects of AKI and, thus, propose melatonin as a pharmacological and therapeutic agent for the inhibition of ferroptosis in AKI.

We performed MTEC RNA-sequence analysis to elucidate the molecular mechanisms that may support the protective effects of melatonin against ferroptosis. KEGG pathway enrichment analysis detected higher enrichment of glutathione metabolism and ferroptosis-associated pathways over that of other programmed cell death pathways in response to melatonin. Our analysis of ferroptosis-related genes showed that the expression of *Slc7a11* was significantly upregulated and the expression of *NRF2* was significantly downregulated after HR treatment, but that melatonin therapy restored their protein expression. NRF2 is an important regulator of the antioxidant system that controls the expression of a series of signal proteins and enzymes by targeting their gene and maintaining redox homeostasis. *Slc7a11*, *glutathione*, and *GPX4* are downstream target genes of NRF2 that are responsible for preventing lipid peroxidation and thus, inhibiting ferroptosis. Li et al. reported that inhibition of ferroptosis by upregulating NRF2 expression delayed the progression of diabetic nephropathy [42], and Yang et al. demonstrated that dimethyl fumarate inhibited ferroptosis by acting on NRF2/GPX4 axis to attenuate acute kidney injury [7]. Based on our RNA-sequence analysis, we hypothesized that melatonin inhibited ferroptosis through NRF2 regulation of the Slc7a11 axis to alleviate AKI. First, we used chromatin immunoprecipitation (ChIP) to validate the interaction of NRF2 and Slc7a11 and found that in melatonin-incubated cells, NRF2 binding to the *Slc7a11* promoter significantly enhanced its promoter activity. When we knocked down *NRF2* in cells, the expression of *Slc7a11* significantly increased and the expression of *GPX4* significantly decreased in the HR group; however, both Slc7a11 and GPX4 proteins could not be restored by melatonin therapy in the absence of *NRF2*. Lastly, in *NRF2* siRNA cells treated with HR, the lipid peroxidation and iron accumulation increased, and melatonin treatment failed to significantly prevent ferroptosis in these cells.

In conclusion, our study revealed that melatonin alleviates AKI by inhibiting ferroptosis via NRF2. The present work provides a new potential for melatonin therapy in the prevention and treatment of AKI.

## Figures and Tables

**Table 1:** Primer sequences used in real-time PCR.

	Forward	Reverse
*KIM-1*	CAGGGAAGCCGCAGAAAA	GAGACACGGAAGGCAACCAC
*IL-1β*	GACTTCACCATGGAACCCGT	CAGGGAGGGAAACACACGTT
*TNF-α*	CATCTTCTCAAAATTCGAGTGACAA	TGGGAGTAGACAAGGTACAACCC
*MCP-1*	CTTCTGGGCCTGCTGTTCA	CCAGCCTACTCATTGGGATCA
*IL-6*	CCACTTCACAAGTCGGAGGCTTA	TGCAAGTGCATCATCGTTGTTC
*PGC-1a*	TATGGAGTGACATAGAGTGTGCT	GTCGCTACACCACTTCAATCC
*TFAM*	GAAACGCCTAAAGAAGAAAGCA	CTGACTCATCCTTAGCCTCCTG
*Acsl4*	ACTTACCTTTGGCTCATG	CAGTACAGTACAATCACCCT
*Cox-2*	CACACTCTATCACTGGCACC	TCCAGGAGGATGGAGTTGTT
*Slc7a11*	GGCACCGTCATCGGATCAG	CTCCACAGGCAGACCAGAAAA
*GPX4*	AGGCAGGAGCCAGGAAG	CCTTGGGCTGGACTTTC
*NRF2*	CTTTAGTCAGCGACAGAAGGAC	AGGCATCTTGTTTGGGAATGTG
*β-Actin*	AGTGTGACGTTGACATCCGT	TGCTAGGAGCCAGAGCAGTA

**Table 2:** Nfe2l2-siRNA.

	Sequences (5′-3′)	
*Nfe2l2(m)-si-1*	Sense	CCGAAUUACAGUGUCUUAA TT
	Antisense	UUAAGACACUGUAAUUCGG TT
*Nfe2l2(m)-si-2*	Sense	CUCGCAUUGAUCCGAGAUA TT
	Antisense	UAUCUCGGAUCAAUGCGAG TT
*Nfe2l2(m)-si-3*	Sense	CAAGGAGCAAUUCAAUGAA TT
	Antisense	UUCAUUGAAUUGCUCCUUG TT
*siRNA NC*	Sense	UUCUCCGAACGUGUCACGU TT
	Antisense	ACGUGACACGUUCGGAGAA TT
